# Effect of the presence and location of corpus luteum on competence of bovine cumulus-oocyte complexes

**DOI:** 10.1590/1984-3143-AR2021-0074

**Published:** 2022-05-06

**Authors:** Edgar Ricardo Moreno Jerez, Alejandra Arias García, Marina Caccia, Aldemar Chávez Rodríguez, Silvia Juliana Restrepo Gonzales, Edgar Mauricio Mogollón Waltero, Diego Fernando Dubeibe Marín

**Affiliations:** 1 Facultad de Medicina Veterinaria y Zootecnia, Universidad Cooperativa de Colombia, Bucaramanga, Santander, Colombia; 2 Empresa Colombiana de Productos Veterinarios – VECOL, Bogotá, Cundinamarca, Colombia; 3 Universidad Nacional de Villa María, Córdoba, Argentina; 4 Private Practice, Bogotá, Cundinamarca, Colombia; 5 Fundación para el Fomento e Investigación de la Producción Sostenible de Alimentos en Santander – FOINPROSAS, Bucaramanga, Santander, Colombia; 6 Facultad de Ciencias Agropecuarias, Universidad de Ciencias Aplicadas y Ambientales – UDCA, Bogotá, Cundinamarca, Colombia

**Keywords:** oocyte competence, sperm penetration, nuclear maturation, bovine embryos

## Abstract

This study aimed to determine the effect of presence of the corpus luteum (CL) and its influence on cumulus–oocyte complexes (COCs) obtained from the ipsilateral or contralateral ovary in bovine on the recovery and capacity of the oocytes to sustain mono-spermic fertilization, undergo preimplantation development, and develop to the blastocyst stage. Ovaries were collected at a local slaughterhouse and kept in pairs corresponding to the same animal. In the first experiment the variables evaluated were compared between cows with (CCL^+^) and without (CCL^-^) CL, and for the second experiment, comparisons were made between ovaries with an ipsilateral (CL^+^), contralateral (CL^−^), and no (NCL). The recovery rate of COCs was higher in ovaries from CCL^−^ cows, and a higher proportion of grade 1 COCs were recovered from this group. A higher proportion of metaphase I oocytes at 7 h of maturation, and a higher rate of cleavage were observed in the CCL^+^ group; however, a higher proportion of embryos were obtained from the CCL^−^ group. Besides, COCs from the CL^+^ group had a lower proportion of grades 1 and 2 morphological qualities, lower rate of metaphase II oocytes at 22 h of maturation, and lower rate of formation of two pronuclei, whereas a higher proportion of unfertilized oocytes after in vitro fertilization. On the other hand, the COCs from the CL^−^ group displayed a lower proportion of oocytes with more than two pronuclei, higher cleavage rate, and higher final blastocyst production were obtained when compared to CL+. Thus, the effects of CL on the competence of bovine COCs are different depending on the anatomical proximity of their location in the animal, negatively affecting the quality of COCs located in the same ovary, but not having negative effects on the competence of COCs in the ovaries contralateral to their location.

## Introduction

In the last 20 years, the number of transferable bovine embryos produced *in vitro* has shown an increase of almost 25 times (Viana, 2020). Some advantages such as the possibility of embryo production without the need for prior hormonal protocols, the adequate efficiency in the use of sexed semen, and the high frequency by which embryos can be produced from the same animal, have turned this technique an attractive, reliable, and affordable tool in cattle industry (Ferré et al., 2020).

Despite advances, the *in vitro* production of bovine embryos shows a high variability of results, and the average production of blastocysts by OPU sessions remains relatively low (approximately 30%–40%) (Baruselli et al., 2015; Ferré et al., 2020). The heterogeneity in the competence of oocytes used as raw material for the production of embryos contributes to the variability of the technique because this characteristic determines the response to maturation *in vitro* and limits the rate of embryonic development to the blastocyst stage (Lonergan and Fair, 2008, 2016). Various factors inherent to donor cows, such as their nutritional status (Sales et al., 2015), age (Yamamoto et al., 2010), stage of the estrus cycle (Machatková et al., 1996; Pirestani et al., 2011), and hormone levels (Fair and Lonergan, 2012), can affect the further oocytes development.

Particularly, the presence of the corpus luteum (CL) at the time of OPU has been the subject of research. The presence of the CL at the time of oocyte collection and/or the factors produced by this transient gland affect the competence of the recovered structures (Moreno et al., 1993; Sugulle et al., 2008; Hajarian et al., 2016; Quezada-Casasola et al., 2018). However, the findings reported in literature show contradictory results. Some studies affirm that presence of a CL exerts a beneficial effect on oocyte population and quality through the local effects of progesterone and growth factors that decrease apoptosis in follicular cells and exert potential beneficial effects on follicular development and oocyte competence (Penitente-Filho et al., 2014, 2015). In addition, due to the increase in blood flow to the ovary containing the CL, the presence of a CL causes an increase in the arrival of nutritive substances such as lipids, proteins, and carbohydrates, besides hormones and growth factors, reaching the ovarian cortex (Argudo et al., 2020). It has been proposed that physical interactions between the follicles and between the CL and the follicles could affect the follicular growth dynamics and ovulation patterns (Ginther, 2020). Thus, for instance, the presence of CL ipsilaterally to the dominant follicle increases the odds for ovulations in that ovary, and this positive relation could be associated with an increased blood flow, favoring the follicle development (Ginther and Dangudubiyyam, 2018).

In contrast, some authors suggested that the CL causes deleterious effects on the follicular population and oocyte quality due to the local production of inhibin by the CL and increased blood flow directed mainly to the luteal tissue, therefore restricting nutritional supply to the remaining structures present in the ovary (Hajarian et al., 2016). On the other hand, some studies indicate that the beneficial or deleterious effects of the CL are not common to all follicular structures present in the same ovary, and small- or medium-sized follicles that are in the growth phase would be mostly harmed by the local inhibitory factors from the CL, possibly related to inhibin or high local concentration of progesterone, whereas in large follicles this effect would not be important (Karami et al., 2015). In contrast, others suggest that presence of the CL presents no observable effects on oocyte quantity and/or quality (Wit et al., 2000; Sugulle et al., 2008).

Thus, the true effect of presence of the CL on the competence of cumulus–oocyte complexes (COCs) has not been completely elucidated. Similarly, the pathways and mechanisms by which the CL exerts its deleterious or beneficial effects are not understood and are mostly based on hypotheses that need to be tested. Therefore, this study aimed to investigate the effect of the presence of the CL and its influence on COCs obtained from the ipsilateral or contralateral ovary on the recovery rate of COCs as well as their maturation and embryonic development competence under *in vitro* conditions.

To evaluate the objective described above, hypothesis tested in this study were: (i) Cows bearing a CL with functional appearance produce more COCs with a greater competence in comparison to cows without CL, and (ii) effects of the CL on COCs quantity and competence are different depending on its ipsilateral or contralateral origin according to the ovary.

## Methods

This study was conducted in the Animal Reproduction Laboratory of the Faculty of Veterinary Medicine and Zootechnics at the Universidad Cooperativa de Colombia, located in the Guatiguará Agricultural Academic Center; the center is located in the municipality of Piedecuesta (Santander, Colombia). All the experimental procedures carried out in this study were approved by the bioethical committee of the Universidad Cooperativa de Colombia (Protocol N°: 024).

### Collection and culture of cumulus-oocyte complexes

Bovine ovaries were collected from a local slaughter plant, separated into pairs corresponding to the same animal, and transported to the laboratory in physiological saline solution [0.9% sodium chloride (NaCl)] supplemented with antibiotics [penicillin G (100 IU/mL)/streptomycin sulfate (100 µg/mL)], at room temperature (~25°C). It took less than two hours from the collecting in the slaughterhouse to the processing of the ovaries in the laboratory. In a period of five months, 20 ovary collections (10 by experiment) were carried out and, 572 ovaries, corresponding to 286 cows were used for this study. The age, breed, and productive and reproductive characteristics of the animals at the time of slaughter were unknown. Ovaries were processed by the same technician along the experiments.

Once in the laboratory, the ovaries were washed in a new 0.9% physiological saline (NaCl) solution at 37°C. The COCs were recovered by follicular aspiration using an 18G × 1^1/2^ gauge needle attached to a 5 mL syringe. Subsequently, they were classified into four different quality grades according to the appearance of cumulus cells and oocyte cytoplasm as described by Leibfried and First (1979). For the culture, the COCs of quality grades 1 and 2 were initially washed in a TCM/HEPES (Sigma®, St Louis, USA) solution, supplemented with 5% fetal bovine serum (FBS) and antibiotics (50 µL/mL streptomycin and 50 IU/mL penicillin), then distributed into groups of 20 COCs, and cultured in drops of 100 µL in TCM-199 medium (Sigma®, St Louis, USA), supplemented with 22 µg/mL sodium pyruvate, 4 mg/mL glutamine, 3 mg/mL ascorbic acid, 0.2 mg/mL myoinositol, 50 µl/mL streptomycin, 50 IU/mL penicillin, 100 mM cysteamine, 2.2 mg/mL sodium bicarbonate, 0.05 IU/mL FSH/LH (Menopur®, Ferring, Wittland, Germany), and 10% FBS. The COCs were cultured at 38.5°C and 95% humidity with 5% CO_2_ for 22 h.

### Experimental design

#### Experiment 1: Evaluation of the recovery and competence of COCs obtained from cows with and without a CL and without a follicle > 10 mm

The COCs were divided into the following two experimental groups: 1) COCs from animals with a CL of functional appearance (i.e., CL embracing more than the 40% from the total volume of the ovary, visible vasculature on its surface, and external and internal yellowish or orange color) in one of the pair of ovaries (CCL+) and 2) COCs obtained from ovaries of cows without a CL neither follicles larger than 10 mm (CCL-). The number of recovered COCs, morphological classification of COCs, kinetics of nuclear maturation, cleavage rate, and blastocyst production were evaluated. This experiment was repeated 10 times in a period of 2 months. For this experiment, 246 ovaries (from 123 cows) were processed in CCL+ group, and 38 ovaries (from 19 cows) in CCL- group.

#### Experiment 2: Effect of the presence and localization of the CL on the recovery and competence of COCs

The effect of the presence of the CL was evaluated considering its presence and its influence on COCs obtained from the ipsilateral or contralateral ovary. The obtained COCs formed the following three experimental groups: 1) COCs from ovaries with a CL (CL+), 2) COCs from ovaries without a CL, but from cows with a CL in the contralateral ovary (CL-), and 3) COCs from animals without a CL and without a follicle > 10 mm (NCL). The following variables were analyzed: recovery rate, morphological classification, nuclear maturation, sperm penetration, cleavage and blastocyst production. This experiment was also repeated 10 times in a period of 3 months. A total of 123, 123 and 42 ovaries were processed in groups CL+, CL- and NCL, respectively.

### *In vitro* production of embryos

After maturation, the COCs were cocultured with 2 × 10^6^ sperm/mL from the same sire and batch, that were previously selected by centrifugation in Percoll gradients (90%, 60%, and 30%) (Matás et al., 2011) for 3 min at 810 x g (Minispin®, Eppendorf, USA) and washed in TALP solution through second centrifugation at 400 x g for 45 sec. *In vitro* fertilization (IVF) was performed in 90 µL drops of IVF-SOF medium, supplemented with 20 mg/mL heparin, 0.18 mM/mL penicillamine, 0.1 mM/mL hypotaurine, and 0.018 mM/mL epinephrine, in an incubator set at 38.5°C, with 95% humidity and 5% CO_2_ for 18 h. The presumptive zygotes were cocultured with cumulus cells for 8 days in 100 µL drops of TCM-199 medium, supplemented with 4.4 mg/mL sodium bicarbonate, 10% FBS, 100 IU/mL penicillin, 100 µl/mL streptomycin, 100 mM sodium pyruvate, and 100 µM cysteamine, under the same conditions described for maturation and IVF. About 50% volume of the culture medium was changed on days 3 and 5, and the cleavage percent and blastocyst production were assessed on days 3 and 8, respectively.

### Evaluation of nuclear maturation

Groups of 10 COCs from each experimental group (CCL+ and CCL- in experiment 1 and, CL+, CL- and NCL in experiment 2) were analyzed at 7, 14, and 22 h of incubation. Initially, the oocytes were mechanically denuded by repeated pipetting in 500 µL PBS solution supplemented with 0.1% PVA. Once washed, the oocytes were mounted between the slide and laminin and fixed in a solution comprising acetic acid and 90% ethanol in a 1:3 ratio for 24 h at 5°C. Subsequently, the oocytes were stained with 2% acetic–orcein (Prentice-Biensch et al., 2012) and evaluated under optical microscope (Zeiss ®, Jena, Germany) at 400X. According to chromatin configuration, oocytes were classified into one of the following meiosis phases: germinal vesicle (GV), germinal vesicle breakdown (GVB), metaphase I (MI), anaphase I (ANA I), telophase I (TEL I), and metaphase II (MII).

### Evaluation of sperm penetration

After 18 h of IVF, groups of 10 presumptive zygotes from each experimental group (CCL+ and CCL- in experiment 1 and, CL+, CL- and NCL in experiment 2) were mechanically denuded, mounted between slide and laminin, fixed in the acetic acid and ethanol solution (1:3 ratio) for 24 h, and subsequently placed in 90% ethanol for 20 min. The structures were stained with Lacmoide stain and evaluated under a microscope at 400X. According to the chromatin characteristics, zygotes with two pronuclei were considered normal, whereas those with more than two pronuclei were considered polyspermic, and in the absence of pronuclei, they were considered to be unfertilized.

### Statistical analysis

For comparations between groups in experiments 1 and 2, adjustments were made using Benjamini–Hochberg at the 5% significance level. The *t*-test was used to compare the number and quality of COCs recovered per ovary in experiment 1 (CCL+ vs CCL-), and a Gaussian mixed-effects model was used to evaluate these results in experiment 2 (CL+, CL- and NCL). Nonparametric Kruskal–Wallis test was used to evaluate the rate of nuclear maturation and sperm penetration. Comparations between experimental groups were made using Wilcoxon Mann–Whitney’s test. For the analysis of cleavage rates and blastocyst production, evaluations were performed with the generalized binomial mixed model, considering the experimental groups as fixed effects and, as random effects the battery of structures used for in vitro fertilization. The analyses were performed using R software version 3.3.1.

## Results

### Experiment 1: Evaluation of the recovery and competence of COCs obtained from cows with and without a CL and without a follicle > 10 mm

In this experiment, comparisons were made between COCs collected from cows with a CL (CCL+) and cows without CL (CCL-) groups.

The average number of COCs classified into quality grade 1 was greater in the ovaries of CCL^−^ cows than in the ovaries of CCL^+^ cows (Table 1), and the average number of recovered COCs was lower in ovaries from CCL^+^ group in relation to CCL^−^ (Table 2).

**Table 1:** Morphological quality of cumulus-oocytes complexes (COCs) recovered per ovary from cows with (CCL^+^) or without a CL (CCL^−^).

**Quality grades**	**CCL^+^**	**CCL^−^**
1	3.54 ± 0.36^a^	7.79 ± 1.44^b^
2	4.02 ± 0.47	8.82 ± 2.13
3	4.19 ± 0.58	6.38 ± 1.63
4	4.46 ± 0.81	6.62 ± 1.30
Total of structures	3815	1067

**Table 2:** Rate of cleavage and blastocyst production from oocytes obtained from cows with (CCL^+^) or without a CL (CCL^−^).

**Groups**	**No. of ovaries**	**No. of COCs**	**Oocytes/ovary**	**Oocytes taken to IVM**	**Cleavage (%)**	**Blastocysts/fertilized oocytes (%)**	**Blastocysts/cleaved embryos (%)**
CCL^+^	246	3815	15.50 ± 1.70 ^b^	1860	57.9 ± 3.6^a^	18.4 ± 2.5^a^	33 ± 5.1^a^
CCL^−^	38	1067	28.07 ± 5.36 ^a^	632	48.9 ± 3.9^b^	20.1 ± 3.1^b^	45 ± 8.7^b^

The values indicate results from seven repetitions. Cleavage and blastocyst rates were assessed just from oocytes with quality grades 1 and 2. Different letters in the same column indicate significant differences (p < 0.05) between groups. COCs: cumulus-oocyte complexes; IVM: in *vitro* maturation; CL: corpus luteum.

Regarding the evaluation of nuclear maturation, at 7 h of culture, no significant difference was observed in the percentage of maturation between the experimental groups (CCL+ vs CCL^−^) when the GV and GVB stages were compared (Table 3). However, at this time of evaluation, a greater proportion of oocytes in the MI stage was observed in the CCL^+^ group than in the CCL^−^ group. No difference was observed between the groups in other stages of nuclear maturation at subsequent evaluation times.

**Table 3:** Percentage of oocytes in each state of meiosis evaluated at 7, 14, and 22 h of *in vitro* culture, from cows with (CCL^+^) and without a CL (CCL^−^).

**State of meiosis**	**7 h**		**14 h**		**22 h**	
**GV**	26.5 ± 5.7	36.2 ± 13.3	1.3 ± 0.8	7.2 ± 5.8		
**GVB**	52.7 ± 2.7	58.6 ± 11.8	3.9 ± 1.9	3.4 ± 2.1		
**MI**	20.6 ± 4.7^a^	5.2 ± 3.4^b^	68.8 ± 2.5	56.2 ± 9.2	12.4 ± 3.2	14.5 ± 8.9
**ANA I**			13.9 ± 4.6	15.7 ± 4.1	4.4 ± 1.8	3.6 ± 2.3
**TEL I**			11.8 ± 3.2	17.4 ± 3.3	25.2 ± 5.9	20.5 ± 10.1
**MII**					57.9 ± 3.2	61.3 ± 12
**Total of structures**	160	82	150	70	188	93

Averages ± standard error, (seven repetitions). Different letters in the same row for each maturation time indicate a significant difference (p < 0.05) between groups. Were evaluated 498 and 245 oocytes from cows with corpus luteum (CCL^+^) and cows without corpus luteum (CCL^−^), respectively. GV, germinal vesicle; GVB, germinal vesicle breakdown; MI, metaphase I; ANA I, anaphase I; TEL I, telophase I; MII, metaphase II. Average ± standard error (SE) of the number of COCs distributed among the four established quality categories. Different letters in the same row indicate a significant difference (p < 0.05). Oocytes recovered from 284 ovaries, corresponding to 142 slaughtered cows. For data collection, the experiment was repeated 10 times. CL: corpus luteum.

On the other hand, even when the percentage of cleaved embryos was greater in the CCL^+^ group, the final blastocyst rate, calculated by the matured oocytes and cleaved embryos, was greater in the group CCL^−^ (Table 2).

### Experiment 2: Effect of the presence and localization of the CL on the recovery and competence of COC

Regarding the morphological quality, the average number of COCs classified into quality grades 1 and 2 recovered from CL^+^ ovaries was lower than that in the other groups (Table 4). The average number of COCs/ovaries recovered from the NCL group was greater than those recovered from the CL^−^ and CL^+^ groups (Table 5), whereas no difference in recovery was observed between the other two groups.

**Table 4:** Morphological quality of COCs recovered per ovary, from ovaries with an ipsilateral (CL+), contralateral (CL−) or no CL in both ovaries (NCL).

**Quality grades**	**CL^−^**	**CL^+^**	**NCL**
1	3.69 ± 0.39^a^	3.38 ± 0.39^b^	7.01 ± 1.58^a^
2	4.33 ± 0.42^a^	3.70 ± 0.42^b^	8.82 ± 2.13^a^
3	3.84 ± 0.85	4.54 ± 0.85	6.38 ± 1.63
4	3.88 ± 0.9	5.04 ± 0.91	5.97 ± 1.40
Total of structures/ovaries	1844/123	1971/123	1067/38

Average ± standard error (SE) of the number of cumulus–oocyte complexes (COCs) distributed among the four established quality categories. Different letters in the same row indicate a significant difference (p < 0.05). Oocytes recovered from 288 ovaries, corresponding to 144 slaughtered cows. For data collection, the experiment was repeated 10 times. CL: corpus luteum.

**Table 5:** Cleavage and blastocyst production rate from COCs obtained from ovaries with an ipsilateral (CL+), contralateral (CL−) or no CL in both ovaries (NCL).

**Groups**	**No. of ovaries**	**No. of COCs**	**Oocytes/ovary**	**Oocytes taken to IVM**	**Cleavage (%)**	**Blastocysts/fertilized oocytes (%)**	**Blastocysts/cleaved embryos (%)**
**CL^−^**	123	1844	15.76 ± 1.79^b^	980	62.2 ± 3.8^a^	21 ± 3.28^a^	35 ± 6.96^a^
**CL^+^**	123	1971	16.67 ± 1.88^b^	460	53.5 ± 3.94^b^	16 ± 2.79^b^	30 ± 4.67^ab^
**NCL**	38	1067	29.62 ± 5.36^a^	350	48.9 ± 3.88^b^	20 ± 3.09^b^	45 ± 8.78^b^

The values indicate results from seven repetitions. Just COCs with grades 1 and 2 were taken for *in vitro* embryo production. Different letters in the same column indicate significant differences (p < 0.05) between groups. COCs: cumulus–oocyte complexes; IVM: *in vitro* maturation; CL: corpus luteum.

The evaluation of oocyte nuclear maturation at 7 h did not show significant differences between the groups (Table 6); however, at 14 h of culture a greater proportion of oocytes in MI was observed in the CL^+^ group than in the NCL group, and the same was true for the oocytes in TEL 1 in the NCL group when compared to the CL^+^ group. At 22 h, the percentage of oocytes in MII was greater in the CL^−^ group than in the CL^+^ group. No differences were observed between the other groups at these last two evaluation times.

**Table 6:** Percentage of oocytes at each stage of meiosis evaluated at 7, 14, and 22 h of *in vitro* maturation, obtained from ovaries with an ipsilateral (CL+), contralateral (CL−) or no CL in both ovaries (NCL).

**Meiosis state**	**7 h**			**14 h**			**22 h**		
**GV**	40.1 ± 9.3	12.9 ± 5.1	31 ± 12.4	1.2 ± 1.2	1.4 ± 1.4	7.2 ± 4.1			
**GVB**	48.9 ± 6.2	56.5 ± 6.4	59.1 ± 9.9	5.1 ± 3.4	2.8 ± 2.8	3.4 ± 1.6			
**MI**	10.8 ± 6.6	30.5 ± 6.7	9.8 ± 5.4	73.3 ± 5.4**^a^**	64.4 ± 5.3**^ab^**	56.2 ± 9.6**^b^**	18.5 ± 6.2	6.3 ± 2.9	14.5 ± 8.9
**ANA I**				13.7 ± 3.9	14.3 ± 6.7	16.3 ± 3.4	6.3 ± 2.8	3.4 ± 2.3	3.6 ± 2.3
**TEL I**				6.7 ± 2.7**^b^**	16.9 ± 5.6**^ab^**	27.8 ± 7.1**^a^**	33.4 ± 8.5	17.1 ± 6.4	20.5 ± 10.1
**M II**							45.6 ± 2.3**^b^**	73.2 ± 6.4**^a^**	61.3 ± 12**^ab^**
**Total of structures**	75	84	80	82	76	75	82	99	90

Averages ± standard error, (seven repetitions). Different letters in the same row for each maturation time indicate significant differences (p < 0.05). A total of 239, 259, and 245 oocytes per experimental group underwent three treatments, CL-, CL^+^, and NCL, respectively; an average of 12.3 oocytes were evaluated by collection, maturation time, and experimental group. GV, germinal vesicle; GVB, germinal vesicle breakdown; MI, metaphase I; ANA I, anaphase I; TEL I, telophase I; MII, metaphase II.

When evaluating the sperm penetration rate, the CL^−^ and NCL groups showed a greater normal pronucleus formation rate (two pronuclei) than the CL^+^ group (Table 7). Similarly, the highest percentage of unfertilized oocytes was observed in the CL^+^ group, and the number of oocytes with more than two pronuclei (polyspermia) was significantly lower in the CL^−^ group than in other groups.

**Table 7:** Sperm penetration rate of oocytes from the experimental groups: ovaries with an ipsilateral (CL+), contralateral (CL−) or no CL in both ovaries (NCL).

**Experimental group**	**2 PN**	**>2 PN**	**UF**	**Total of structures**
**NCL**	68.4 ± 8.6 ^a^	13.2 ± 4.5 ^a^	18.4 ± 4.7 ^c^	40
**CL^+^**	47.5 ± 5.3 ^b^	13.2 ± 4.4 ^a^	39.2 ± 6.5 ^a^	70
**CL^−^**	63.8 ± 5.9 ^a^	7.6 ± 3.8 ^b^	28.5 ± 8.5 ^b^	70

Average ± standard error of the number of cumulus–oocyte complexes evaluated at 18 h of IVF culture and classified according to the formation of pronuclei into 2 PN: two pronuclei or normal fertilization, >2 PN: more than two pronuclei or polyspermics; UF: unfertilized oocytes. Oocytes were obtained from seven experimental repetitions. Different letters in the same column indicate significant differences (p<0.05).

Finally, the cleavage rate was greater in the CL^−^ group embryos than in the CL^+^ and NCL groups (Table 5), whereas no differences were observed between the CL^+^ and NCL groups. In the same way, the percentage of blastocyst production calculated from the oocytes placed in the fertilizing medium was greater in the CL^−^ group than in the CL^+^ and NCL groups, and the evaluation of the blastocyst rate calculated by cleaved embryos was greater in the CL^−^ group than in the NCL group, but it was not different from the CL^+^ group.

## Discussion

To determine the effect of the presence of the CL on the competence of bovine COCs, two independent experiments were conducted. In the first experiment, the effect of the CL was evaluated by taking into account its presence or absence in oocytes collected from slaughterhouse animals. The recovery rate and morphological quality of the COCs, in addition to the blastocyst production were found to be higher in the group of COCs obtained from cows without a CL and without a follicle > 10 mm. These results are consistent with other studies that reported a negative effect of presence of a CL on the follicular population and oocyte competence (Karami et al., 2015; Hajarian et al., 2016). The mechanisms associated with this effect have not been completely identified; however, it is hypothesized that the CL produces considerable amount of inhibin locally that interferes with follicular development and consequently with the quality of the COCs present within (Hajarian et al., 2016). Additionally, the blood flow in ovaries with the CL would be prioritized over the luteal tissue, thus restricting nutrient supply to the remaining structures present in the ovarian cortex (Shabankareh et al., 2010).

In contrast, Saad et al. (2019) compared the recovery rate of COCs between cows classified into cyclic (with a CL) and acyclic (without a CL) and obtained a higher number of COCs in cyclic cows, disagreeing with the results obtained in this study. However, other factors such as the genetic of the slaughtered animals, this is, differences in follicular population between *Bos Indicus* and *Bos Taurus* breeds (Guerreiro et al., 2014), in addition to differences in metabolic and nutritional status of animals, affecting the follicular development (Diskin et al., 2003), and even the skill of the operator who performs the follicular aspiration (Merton et al., 2003) can affect the recovery rate of the structures, which could explain the differences observed between these studies. Due to the origin of the samples for this experiment, being collected from a slaughterhouse, the information about animals is limited and other factors that could influence the follicular development (age, nutritional, gestational, or follicular status) cannot be explained accurately in this study.

The evaluation of the kinetics of nuclear maturation revealed that a greater proportion of oocytes from animals with a CL reached the status of MI at 7 h of culture, and although no significant differences were observed at subsequent evaluation times, this result is consistent with the higher percentage of cleavage observed at day 3 in the embryos produced from the COCs of this group. This shows that although the rate of maturation and the subsequent cleavage was higher, the competence of oocytes, evaluated according to the final rate of blastocysts produced from cows with a CL, was lower. However, considering that these results contradict other findings reported in literature (Gonzalez-Bulnes et al., 2005; Manjunatha et al., 2007) and to further investigate the possible effect of the CL, a second experiment was designed to evaluate the effect of the presence of the CL as well as the effect of its location in the same animal possessing the CL.

It was observed that the CL did not affect the number of COCs recovered from ovaries with ipsilateral (CL^+^) or contralateral (CL^−^) CLs; however, its location was a determining factor in the morphological quality of the COCs because a significantly lower number of COCs of quality grades 1 and 2 were obtained from the group of ovaries with a CL. Additionally, the evaluation of nuclear maturation corroborated the theory that the nuclear competence of oocytes from CL^+^ ovaries was lower than those obtained from the CL^−^ group of ovaries, which was evidenced by a lower number of oocytes in MII at 22 h of culture. These results are consistent with those observed in the evaluation of the sperm penetration rate, in which a lower number of fertilized oocytes with the formation of two pronuclei (normal fertilization) and a higher rate of unfertilized oocytes were obtained in the group of COCs from ovaries with a CL. These results corroborate the findings observed in the first experimental phase of the present study and agree with other authors; however, they are in contrast with what was observed by Penitente-Filho et al. (2014, 2015) and Argudo et al. (2020), who reported better morphological quality and COC competence obtained from ovaries with a CL. Although it is difficult to determine the causes of discrepancies in these observations, they may in part be due to differences in characteristics of ovaries used for experimentation and laboratory proceedings, including factors such as the characteristics of the sacrificed animals (breed and metabolic conditions) from which the ovaries were obtained, and the specific culture conditions used by the different laboratories (composition of the culture media).

In contrast, through the analyses of nuclear maturation, sperm penetration, and blastocyst production, the results of the present study showed that the group of COCs from ovaries without a CL but belonging to cows with a CL in the other ovary, had higher cell competence, which was reflected in higher rates of MII, formation of two pronuclei postfertilization, and final blastocyst rate. Although the mechanisms associated with these effects were not analyzed, it is possible that the COCs present in ovaries without a CL do not suffer the negative effects described above, which is related to their proximity to the follicular structures of the same ovary. However, their location in the same animal who has a CL in the contralateral ovary would allow them benefiting from the action of substances produced by the CL and released into the bloodstream, such as progesterone. It has been associated with a positive effect of progesterone on the quality of recovered oocytes (Saad et al., 2019), and this effect could be mediated mainly by its antiapoptotic activity on follicular cells (Fair and Lonergan, 2012; P. Lonergan and Sánchez, 2020). *In vivo* studies have established its role in proper follicular development because cows who have elevated progesterone levels during the final development of the dominant follicle present with better pregnancy rates after the process of artificial insemination (Bisinotto and Santos, 2011; Wiltbank et al., 2011). The quality of COCs and embryos improved in cattle subjected to superovulation and embryo flushing (Nasser et al., 2011) when subjected to high concentrations of progesterone during the follicular development phase. In addition, high concentrations of progesterone are associated with a greater number of follicular growth waves (Savio et al., 1993; Diaz et al., 1998). Constant follicular turnover could increase the supply of young follicles containing oocytes with high developmental competence. On the other hand, the lack of an active CL and lack of a follicle > 10 mm could be found in anovulatory or recently ovulated cows. In both situations, the number of small follicles and lack of inhibitor factors from a dominant follicle may differ from animals in other stages of the estrous cycle.

## Conclusion

Thus, the experimental approach used in this study made it possible to establish the paradox effect of a CL on the competence of bovine COCs. The dual effect of the CL observed in this study could be partly explained by its location in the animal, negatively affecting the quality of COCs from ovaries with an ipsilateral CL, but not affecting the competence of COCs located in ovaries with a contralateral CL. Because of the difficulty of the obtention of data information (age, breed, ovarian dynamics, gestational status etc.), when using ovaries from slaughterhouse, the results should be interpreted carefully. New experiments, using the same experimental approach but with COCs from live animals will be necessary to confirm these results.

## References

[B001] Argudo DE, Tenemaza MA, Merchán SL, Balvoa JA, Méndez MS, Soria ME, Galarza LR, Ayala LE, Hernández-Fonseca HJ, Perea MS, Perea FP (2020). Intraovarian influence of bovine corpus luteum on oocyte morphometry and developmental competence, embryo production and cryotolerance. Theriogenology.

[B002] Baruselli PS, Vieira LM, Batista EOS, Ferreira RM, Sales JNS, Gimenes LU, Torres-Junior JRS, Martins CM, Sá MF, Bo GA (2015). Updates on embryo production strategies. Anim Reprod.

[B003] Bisinotto RS, Santos JEP (2011). The use of endocrine treatments to improve pregnancy rates in cattle. Reprod Fertil Dev.

[B004] Diaz T, Schmitt EJ, de la Sota RL, Thatcher MJ, Thatcher WW (1998). Human chorionic gonadotropin-induced alterations in ovarian follicular dynamics during the estrous cycle of heifers. J Anim Sci.

[B005] Diskin MG, Mackey DR, Roche JF, Sreenan JM (2003). Effects of nutrition and metabolic status on circulating hormones and ovarian follicle development in cattle. Anim Reprod Sci.

[B006] Fair T, Lonergan P (2012). The role of progesterone in oocyte acquisition of developmental competence. Reprod Domest Anim.

[B007] Ferré LB, Kjelland ME, Strøbech LB, Hyttel P, Mermillod P, Ross PJ (2020). Review: recent advances in bovine in vitro embryo production: reproductive biotechnology history and methods. Animal.

[B008] Ginther OJ, Dangudubiyyam SV (2018). Factors affecting side of ovulation in heifers and mares: a comparative study. Anim Reprod Sci.

[B009] Ginther OJ (2020). Intraovarianism: local mechanisms that affect follicle and luteal dynamics in heifers and women. Biol Reprod.

[B010] Gonzalez-Bulnes A, Berlinguer F, Cocero MJ, Garcia-Garcia RM, Leoni G, Naitana S, Rosati I, Succu S, Veiga-Lopez A (2005). Induction of the presence of corpus luteum during superovulatory treatments enhances in vivo and in vitro blastocysts output in sheep. Theriogenology.

[B011] Guerreiro BM, Batista EOS, Vieira LM, Sá MF, Rodrigues CA, Castro A, Silveira CRA, Bayeux BM, Dias EAR, Monteiro FM, Accorsi M, Lopes RNVR, Baruselli PS (2014). Plasma anti-mullerian hormone: an endocrine marker for in vitro embryo production from *Bos taurus* and *Bos indicus* donors. Domest Anim Endocrinol.

[B012] Hajarian H, Shahsavari MH, Karami-shabankareh H, Dashtizad M (2016). The presence of corpus luteum may have a negative impact on in vitro developmental competency of bovine oocytes. Reprod Biol.

[B013] Karami HK, Shahsavari MH, Hajarian H, Moghaddam G (2015). In vitro developmental competence of bovine oocytes: effect of corpus luteum and follicle size. Iran J Reprod Med.

[B014] Leibfried L, First NL (1979). Characterization of bovine follicular oocytes and their ability to mature in vitro. J Anim Sci.

[B015] Lonergan P, Fair T (2008). In vitro-produced bovine embryos: dealing with the warts. Theriogenology.

[B016] Lonergan P, Fair T (2016). Maturation of oocytes in vitro. Annu Rev Anim Biosci.

[B017] Lonergan P, Sánchez JM (2020). Symposium review: progesterone effects on early embryo development in cattle. J Dairy Sci.

[B018] Machatková M, Jokešová E, Petelíková J, Dvořáček V (1996). Developmental competence of bovine embryos derived from oocytes collected at various stages of the estrous cycle. Theriogenology.

[B019] Manjunatha BM, Gupta PSP, Ravindra JP, Devaraj M, Ramesh HS, Nandi S (2007). In vitro developmental competence of buffalo oocytes collected at various stages of the estrous cycle. Theriogenology.

[B020] Matás C, Vieira L, García-Vázquez FA, Avilés-López K, López-Úbeda R, Carvajal JA, Gadea J (2011). Effects of centrifugation through three different discontinuous Percoll gradients on boar sperm function. Anim Reprod Sci.

[B021] Merton JS, de Roos APW, Mullaart E, de Ruigh L, Kaal L, Vos PLAM, Dieleman SJ (2003). Factors affecting oocyte quality and quantity in commercial application of embryo technologies in the cattle breeding industry. Theriogenology.

[B022] Moreno JF, Flores-Foxworth G, Westhusin M, Kraemer DC (1993). Influence of pregnancy and presence of a CL on quantity and quality of bovine oocytes obtained from ovarian follicles aspirated post-mortem. Theriogenology.

[B023] Nasser LF, Sá MF, Reis EL, Rezende CR, Mapletoft RJ, Bó GA, Baruselli PS (2011). Exogenous progesterone enhances ova and embryo quality following superstimulation of the first follicular wave in Nelore (*Bos indicus*) donors. Theriogenology.

[B024] Penitente-Filho J, Carrascal E, Oliveira F, Zolini A, Oliveira C, Soares Í, Torres C (2014). Influence of dominant follicle and corpus luteum on recovery of good quality oocytes for in vitro embryo production in cattle. Br Biotechnol J.

[B025] Penitente-Filho JM, Jimenez CR, Zolini AM, Carrascal E, Azevedo JL, Silveira CO, Oliveira FA, Torres CAA (2015). Influence of corpus luteum and ovarian volume on the number and quality of bovine oocytes. Anim Sci J.

[B026] Pirestani A, Hosseini SM, Hajian M, Forouzanfar M, Moulavi F, Abedi P, Gourabi H, Shahverdi A, Taqi Dizaj AV, Nasr Esfahani MH (2011). Effect of ovarian cyclic status on in vitro embryo production in cattle. Int J Fertil Steril.

[B027] Prentice-Biensch JR, Singh J, Alfoteisy B, Anzar M (2012). A simple and high-throughput method to assess maturation status of bovine oocytes: comparison of anti-lamin A/C-DAPI with an aceto-orcein staining technique. Theriogenology.

[B028] Quezada-Casasola A, Martínez-Armendáriz KE, Itzá-Ortiz MF, Escárcega-Ávila AM, Pérez-Eguía E, Filipiak Y, Larocca C, Carrera-Chávez JM (2018). Effect of presence of corpora lutea on cumulus expansion of in vitro matured bovine oocytes selected by trypan blue and brilliant cresyl blue tests. J Appl Anim Res.

[B029] Saad M, Sarwar Z, Saleem M, Arshad U, Shahzad M, Hassan Mushtaq M, Husnain A, Riaz A, Ahmad N (2019). Effect of plasma progesterone on oocyte recovery, oocyte quality, and early in-vitro developmental competence of embryos in *Bos indicus* dairy cows. Anim Reprod Sci.

[B030] Sales JNS, Iguma LT, Batista RITP, Quintão CCR, Gama MAS, Freitas C, Pereira MM, Camargo LSA, Viana JHM, Souza JC, Baruselli PS (2015). Effects of a high-energy diet on oocyte quality and in vitro embryo production in *Bos indicus* and *Bos taurus* cows. J Dairy Sci.

[B031] Savio JD, Thatcher WW, Morris GR, Entwistle K, Drost M, Mattiacci MR (1993). Effects of induction of low plasma progesterone concentrations with a progesterone-releasing intravaginal device on follicular turnover and fertility in cattle. J Reprod Fertil.

[B032] Shabankareh HK, Habibizad J, Sarsaifi K, Cheghamirza K, Jasemi VK (2010). The effect of the absence or presence of a corpus luteum on the ovarian follicular population and serum oestradiol concentrations during the estrous cycle in Sanjabi ewes. Small Rumin Res.

[B033] Sugulle AH, Dochi O, Koyama H (2008). Developmental competence of bovine oocytes selected by brilliant cresyl blue staining: effect of the presence of corpus luteum on embryo development. J Mamm Ova Res.

[B034] Viana JHM (2020). 2019 statistics of embryo production and transfer in domestic farm animals. Divergent trends for IVD and IVP embryos. Embryo Technology Newsletter..

[B035] Wiltbank MC, Souza AH, Carvalho PD, Bender RW, Nascimento AB (2011). Improving fertility to timed artificial insemination by manipulation of circulating progesterone concentrations in lactating dairy cattle. Reprod Fertil Dev.

[B036] Wit AA, Wurth YA, Kruip TA (2000). Effect of ovarian phase and follicle quality on morphology and developmental capacity of the bovine cumulus-oocyte complex. J Anim Sci.

[B037] Yamamoto T, Iwata H, Goto H, Shiratuki S, Tanaka H, Monji Y, Kuwayama T (2010). Effect of maternal age on the developmental competence and progression of nuclear maturation in bovine oocytes. Mol Reprod Dev.

